# Remnant cholesterol and the risk of diabetic nephropathy progression to end-stage kidney disease in patients with type 2 diabetes mellitus: a longitudinal cohort study

**DOI:** 10.1007/s12020-024-03948-4

**Published:** 2024-07-12

**Authors:** Yuancheng Zhao, Ke Liu, Yutong Zou, Yucheng Wu, Jia Yang, Xiang Xiao, Xuegui Ju, Qin Yang, Yanlin Lang, Fang Liu

**Affiliations:** 1 Department of Nephrology, West China Hospital of Sichuan University, Chengdu, 610041 China; 2 Laboratory of Diabetic Kidney Disease, Kidney Research Institute, Department of Nephrology, West China Hospital, Sichuan University, Chengdu, 610041 China

**Keywords:** Remnant cholesterol, Type 2 diabetes mellitus, Diabetic nephropathy, End-stage kidney disease

## Abstract

**Aim:**

Diabetic nephropathy (DN) is the most common cause of end-stage kidney disease (ESKD). Remnant cholesterol has been investigated as a predictor for the progression of DN in type 1 diabetes mellitus patients, as well as the incidence of DN in type 2 diabetes mellitus (T2DM) patients. This study aimed to evaluate the longitudinal relationship between baseline remnant cholesterol and kidney outcomes using a Chinese T2DM with biopsy-confirmed DN cohort.

**Methods:**

We included 334 patients with T2DM and biopsy-confirmed DN during 2010–2019 West China Hospital T2DM-DN cohort. Remnant cholesterol was defined by Martin-Hopkins equation. Patients were divided into four groups based on the median (IQR) remnant cholesterol concentration at the time of renal biopsy. The kidney outcome was defined as ESKD, which was defined as the need for chronic kidney replacement therapy or estimated glomerular filtration rate (eGFR) < 15 mL/min/1.73 m^2^. The relationship between remnant cholesterol and kidney outcome was analyzed using the Kaplan‒Meier method and Cox regression analysis.

**Results:**

The mean age was 51.1 years, and 235 (70%) were men. During follow-up, a total of 121 (36.2%) patients reached ESKD. The Kaplan‒Meier analysis showed that patients in the highest quartile (quartile 4) group had lower cumulative renal survival (log-rank test, p = 0.033) and shorter median renal survival time [34.0 (26.4–41.6) vs. 55.0 (29.8–80.2) months] than patients in the lowest quartile (quartile 1) group. By univariate analysis, the high remnant cholesterol group was associated with a higher risk of progression to ESKD. Moreover, the risk of progression to ESKD in the highest quartile was still 2.857-fold (95% CI 1.305–6.257, p = 0.009) higher than that in the lowest quartile, and one-SD increase of remnant cholesterol was associated with a higher risk (HR = 1.424; 95% CI 1.075–1.886, p = 0.014) of progression to ESKD, after adjusted for confounding factors.

**Conclusions:**

High remnant cholesterol is independently associated with a higher risk of ESKD in patients with T2DM-DN, and it may be a new noninvasive marker of ESKD.

**Clinical relevance:**

Calculated remnant cholesterol has the advantages of being economical and clinically accessible. Moreover, to our knowledge, there are no longitudinal cohort studies for investigating the risk of progression of T2DM-DN to ESKD. In our study, higher remnant cholesterol was associated with a higher risk of ESKD in patients with T2DM-DN, and it may be a new noninvasive predictor of ESKD.

## Introduction

The prevalence of diabetes has gradually increased over the past decades. According to the 10th edition of the IDF Diabetes Atlas [1], in 2021, it is estimated that 537 million people have diabetes and over 6.7 million people aged 20–79 will die from diabetes-related causes. Diabetes not only damages the physical and mental health of people but also causes a great economic burden. Diabetic nephropathy (DN) is one of the most serious microvascular complications of diabetes and the leading cause of end-stage kidney disease (ESKD) in developing and some developed countries [2, 3]. There are some factors that are prognostic markers for DN prognosis, such as proteinuria and estimated glomerular filtration rate (eGFR) [4], however, even after improving many controllable risk factors, patients still have residual risks for ESKD and cardiovascular events.

Dyslipidemia is extremely common in patients with type 2 diabetes mellitus (T2DM). Previous studies have indicated that dyslipidemia is strongly associated with an increased risk of cardiovascular disease (CVD) in patients with T2DM [5]. A growing body of evidence suggests a strong atherogenicity of the cholesterol in triglyceride-rich lipoproteins (TRLs), also termed remnant cholesterol (RC) that is composed of very-low-density lipoproteins (VLDLs) and intermediate-density lipoproteins (IDLs) in the fasting state and of VLDL, IDL and chylomicron remnants in the non-fasting state [6]. Numerous studies have shown that RC is strongly associated with adverse cardiovascular events and mortality in the general population and T2DM patients [7–9]. In addition, recent studies have shown that RC is associated with the incidence of chronic kidney disease (CKD) in the general population [10], the incidence of DN in T2DM patients [11], and the progression of DN and diabetic retinopathy (DR) in type 1 diabetes mellitus (T1DM) patients [12]. However, there are few studies on the relationship between RC and the progression to ESKD in patients with T2DM and biopsy-proven DN.

Therefore, this study aimed to evaluate the relationship between RC and the progression to ESKD in patients with T2DM-DN.

## Method

### Patient selection and study design

**Fig. 1:**
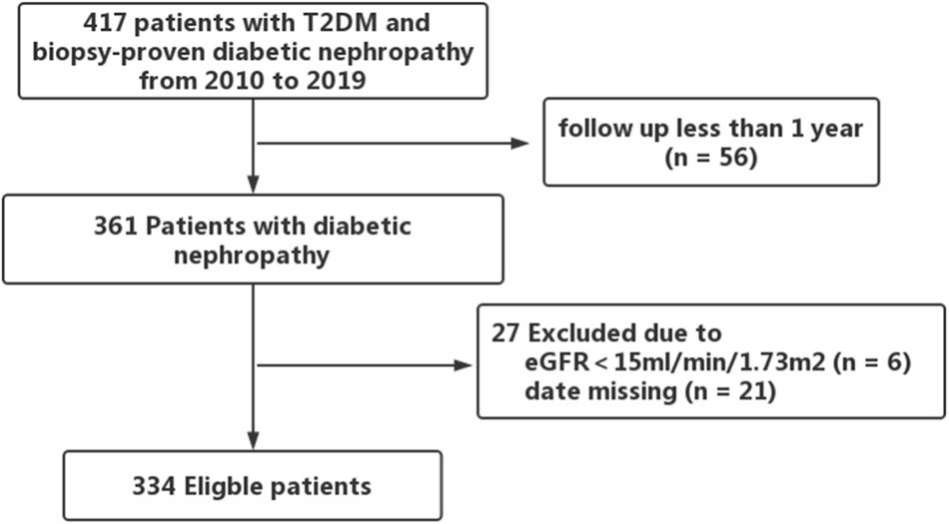
Flowchart of study participants

This study included adult patients with T2DM who underwent kidney biopsy from 2010 to 2019 at the West China Hospital of Sichuan University. All patients were followed up for at least 1 year. The exclusion criteria were as follows: 1) combined with systemic disease and 2) reached ESKD before kidney biopsy (Fig. 1). Diabetes is diagnosed based on American Diabetes Association criteria [13]. The kidney biopsy indicators in T2DM patients are as follows: sudden onset of significant albuminuria, rapid deterioration of renal function (decrease in estimated glomerular filtration rate (eGFR), the eGFR was calculated using the CKD-EPI equation [14], absence of DR, or presence of active urinary sediment. The pathological diagnosis of DN was confirmed by at least two pathologists in the Pathology Department of West China Hospital. The definition of renal pathological features is based on the 2010 Society for Renal Pathology Classification [15]. All patients provided written informed consent, and this study was approved by the institutional review board at the West China Hospital of Sichuan University.

### Clinical and pathological features

Baseline clinical data were obtained by interview and anthropometrics, including sex, age, body mass index, duration of diabetes, blood pressure, dyslipidemia, and medication history. Urine samples and blood samples were obtained using a biochemical automated analyzer (Cobas Intera 400 Plus, Roche, Basel, Switzerland) and included blood creatinine, triglycerides, cholesterol, HbA1c, fasting glucose, and 24-hour urine protein. Triglycerides, total cholesterol, LDL cholesterol, and HDL cholesterol were directly measured using a Triglyceride Assay Kit (GPO-PAP Method), Cholesterol Kit (CHOD-PAP Method), low-density lipoprotein (LDL) cholesterol Kit (Surfactant Assay), and high-density lipoprotein (HDL) Cholesterol Kit (CAT Assay) by a BECKMAN COULTER AU5800 Series Chemistry Analyzer. Dyslipidemia was defined as triglycerides >2.3 mmol/L, total cholesterol ≥6.2 mmol/L, LDL cholesterol >4.1 mmol/l, HDl. cholesterol <1.0 mmol/l, or any lipid lowering medication or self-reported history of dyslipidemia based on the Guidelines on Prevention and Treatment of Dyslipidemia for Chinese Adults [11]. Remnant cholesterol was defined by Martin-Hopkins equation [16], calculation as total cholesterol - LDL cholesterol - HDL cholesterol. Total cholesterol and HDL in the formulas are usually obtained from direct measurements, and LDL can be derived from direct measurements or formulas.

Renal pathology specimens were obtained by needle and evaluated by light microscopy, electron microscopy, and immunofluorescence to assess kidney pathological features, which is known as the Renal Pathology Society classification.

### Kidney outcomes

The kidney outcome was defined by the progression to ESKD, which was defined as the need for chronic kidney replacement therapy or eGFR <15 mL/min/1.73 m^2^ over the 3 months [17].

### Statistical analysis

R software version 4.0.2 was used to perform all statistical analyses. Continuous variables are presented as the mean ± standard deviation or median and quartile range according to normality. Differences between groups were assessed by one-way ANOVA for normally distributed variables and nonparametric tests for skewed distributed variables, such as proportions, and the chi-square test for categorical variables. The correlation test was performed to analyze the associations between RC and DN. Kaplan‒Meier analysis was performed for kidney survival analysis using the log-rank test. Univariate and multivariate Cox analyses were used to examine the relationship between RC and renal outcomes. *p* values < 0.05 were considered to be significant.

## Result

### Baseline clinical and pathological features

A total of 334 patients were included in the study (Fig. 1). At the time of the kidney biopsy, the mean age of the total cohort was 51.1 ± 9.3 years, 70% (235 in 334) were male, 86% (288 in 334) had hypertension, and 48% (159 in 334) had a history of smoking. The mean BMI of patients was 25.6 ± 3.7 kg/m^2^, the mean serum albumin was 34.5 ± 7.9 g/L, and the mean hemoglobin was 119.0 ± 24.1 g/L. The median duration of diabetes in the cohort was 96 (36–132) months, the median eGFR was 60.7 (44.1–92.6) ml/min/1.73 m^2^, and the median urinary protein excretion was 3.8 (1.8–7.0) g/d. Median triglycerides, total cholesterol, LDL-cholesterol, and HDL-cholesterol in the cohort were 1.8 (1.3, 2.4), 5.0 (4.2–6.1), 2.9 (2.2–3.7) and 1.2 (1.0–1.5) mmol/L, respectively. There are 262 (78.4%) had dyslipidemia, including 81 (97.6%) in Quartile 4. A total of 78.7% (263 in 334) of participants had a history of RAAS inhibitor use, and 58.1% (194 in 334) of the participants had a history of statin use. Baseline characteristics are presented in Table 1.

**Table 1:** The baseline clinical features

Parameters	Total n = 334	Quartile 1, n = 84	Quartile 2, n = 86	Quartile 3, n = 81	Quartile 4, n = 83	*P* value
Remnant cholesterol (mmol/L)	0.68 (0.46–1.02)	0.29 (0.21–0.42)	0.59 (0.53–0.65)	0.82 (0.76–0.92)	1.34 (1.17–1.89)	
Age (years)	51.1 ± 9.3	51.7 ± 9.7	51.2 ± 9.5	50.4 ± 9.4	50.9 ± 8.7	0.848
Gender (male, %)	235 (70%)	58 (69.0%)	65 (75.6%)	54 (66.7%)	58 (69.9%)	0.630
Body mass index (kg/m2)	25.6 ± 3.7	25.7 ± 3.6	25.1 ± 3.0	25.8 ± 3.4	25.7 ± 4.6	0.698
Smoking (%)	159 (48%)	44 (52.4%)	42 (48.8%)	36 (44.4%)	37 (44.6%)	0.637
Hypertension (%)	288 (86%)	70 (83.3%)	73 (94.9%)	76 (93.8%)	69 (83.1%)	0.149
Duration of diabetes (months)	96 (36–132)	108 (48–144)	96 (48–132)	72 (36–132)	108 (36–144)	0.346^a^
Fasting blood glucose (mmol/L)	8.4 ± 4.3	8.7 ± 5.6	7.5 ± 3.1	8.1 ± 4.1	9.3 ± 3.9	0.051
HbA1c (%)	7.6 ± 1.9	7.6 ± 1.8	7.6 ± 1.8	7.6 ± 2.2	7.8 ± 1.8	0.891
e-GFR (ml/min·1.73m2)	60.7 (44.1–92.6)	64.3 (46.2–90.3)	68.5 (45.7–97.4)	58.8 (43.3–85.7)	58.0 (42.6–94.6)	0.515^a^
Serum creatinine (umol/L)	115 (82–150)	117 (85–155)	107 (79–150)	117 (82–153)	115 (86–150)	0.854^a^
24 h proteinuria (g/d)	3.8 (1.8–7.0)	4.0 (2.0–7.9)	4.2 (1.6–6.4)	4.9 (2.2–7.7)	3.0 (1.5–6.2)	0.243^a^
Serum albumin (g/L)	34.5 ± 7.9	35.0 ± 7.2	34.3 ± 8.2	34.4 ± 8.2	34.5 ± 8.0	0.948
Hemoglobin (g/L)	119.0 ± 24.1	116.5 ± 22.1	125.1 ± 23.6	117.5 ± 27.6	116.6 ± 21.9	0.058
Triglyceride (mmol/L)	1.8 (1.3–2.4)	1.0 (0.8–1.3)	1.6 (1.3–1.9)	1.8 (1.6–2.3)	3.4 (2.6–4.6)	<0.001^a^
Total cholesterol (mmol/L)	5.0 (4.2–6.1)	4.4 (3.6–5.3)	4.6 (3.9–5.4)	5.1 (4.4–6.2)	6.1 (5.1–7.4)	<0.001^a^
LDL cholesterol (mmol/L)	2.9 (2.2–3.7)	2.6 (1.9–3.3)	2.7 (2.1–3.3)	3.1 (2.3–3.8)	3.4 (2.4–4.2)	0.002^a^
HDL cholesterol (mmol/L)	1.2 (1.0–1.5)	1.5 (1.1–1.8)	1.2 (1.0–1.5)	1.2 (1.0–1.4)	1.0 (0.9–1.2)	<0.001^a^
Dyslipidemia (%)	262 (78.4%)	60 (71.4)	58 (67.4)	63 (77.8)	81 (97.6)	<0.001
RAAS-inhibitor use (%)	263 (78.7%)	69 (82.1%)	68 (79.1%)	60 (74.1%)	66 (79.5%)	0.650
Statin use (%)	194 (58.1%)	52 (61.9%)	44 (51.2%)	46 (56.8%)	52 (62.7%)	0.397
Insulin use (%)	231 (69.2%)	59 (70.2%)	53 (61.6%)	61 (75.3%)	58 (69.9%)	0.285

Data are presented as the mean ± standard, the median or counts, and percentages. Differences between groups were analyzed using the ANOVA, the Kruskal–Wallis H test, or the chi-square test, as appropriate

*eGFR* estimated glomerular filtration rate, *FBS* fasting blood sugar, *RAAS-inhibitor* renin angiotensin aldosterone system inhibitor.

^a^Indicated the use of the Kruskal–Wallis H test

In terms of pathological features, we studied this cohort according to the RPS classification. For the glomerular classification, 15 (4.5%) were in class I, 73 (21.8%) in class IIa, 44 (13.2%) in class IIb, 154 (46.1%) in class III, and 48 (14.4%) in class IV. Most patients had interstitial fibrosis and tubular atrophy (IFTA) (97.3%, 325/334), interstitial inflammation (95.5%, 319/334), arteriolar hyaline degeneration (92.5%, 209/334), and arteriosclerosis (92%, 207/334) in their renal biopsy samples (Table 2).

**Table 2:** The baseline pathological characteristics

Pathological lesions	Total n = 334	Quartile 1, n = 84	Quartile 2, n = 86	Quartile 3, n = 81	Quartile 4, n = 83	*P* value
RPS classification						0.425
I	15 (4.5%)	4 (4.8%)	2 (2.3%)	4 (4.9%)	5 (6.0%)	
IIa	73 (21.8%)	12 (14.3%)	20 (23.3)	21 (25.9%)	20 (24.1%)	
IIb	44 (13.2%)	15 (17.9%)	11 (12.8%)	7 (8.6%)	11 (13.3%)	
III	154 (46.1%)	45 (53.5%)	42 (48.8%)	35 (43.2%)	32 (38.5%)	
IV	48 (14.4%)	8 (9.5)	11 (12.8%)	14 (17.4%)	15 (18.1%)	
IFTA						0.544
0	9 (2.7%)	1 (1.2%)	2 (2.3%)	1 (1.2%)	5 (6.0%)	
1	152 (45.5%)	37 (44.0%)	42 (48.8%)	41 (50.6%)	32 (38.6%)	
2	144 (43.1%)	37 (44.0%)	35 (40.7%)	32 (39.6%)	40 (48.2%)	
3	29 (8.7%)	9 (10.8%)	7 (8.2%)	7 (8.6%)	6 (7.2)	
Interstitial inflammation						0.295
0	15 (4.5%)	3 (3.6%)	4 (4.7%)	2 (2.5%)	6 (7.2%)	
1	247 (74.0%)	58 (69.0%)	70 (81.3%)	59 (72.8%)	60 (72.3%)	
2	72 (21.5%)	23 (27.4%)	12 (14.0%)	20 (24.7%)	17 (20.5%)	
Arteriolar hyalinosis						0.076
0	27 (8.1%)	13 (15.5%)	5 (5.8%)	2 (2.5%)	7 (8.4%)	
1	197 (59.0%)	48 (57.1%)	53 (61.6%)	51 (63.0%)	45 (54.3%)	
2	110 (32.9%)	23 (27.4%)	28 (32.6%)	28 (34.5%)	31 (37.3%)	
Arteriosclerosis						0.358
0	35 (10.5%)	12 (14.3%)	10 (11.6%)	8 (9.9%)	5 (6.0%)	
1	174 (52.1%)	41 (48.8%)	45 (52.4%)	48 (59.2%)	40 (48.2%)	
2	125 (37.4%)	31 (36.9%)	31 (36.0%)	25 (30.9%)	38 (45.8%)	

Correlations between the remnant cholesterol level and histopathological findings. Differences between groups were analyzed using the chi-square test. A two-tailed p < 0.05 was considered statistically significance

*RPS classification* Renal Pathology Society classification, *IFTA* interstitial fibrosis and tubular atrophy

### Relation between RC and clinicopathological features

Based on the median and quartile range of baseline RC concentrations, all patients were divided into four groups: Quartile 1: RC ≤ 0.46 mmol/L; Quartile 2: RC > 0.46 mmol/L and RC ≤ 0.68 mmol/L; Quartile 3: RC > 0.68 mmol/L and RC ≤ 1.02 mmol/L; and Quartile 4: RC > 1.02 mmol/L.

In this cohort, Quartile 1, Quartile 2, Quartile 3, and Quartile 4 had 25.1% (84 in 334), 25.7% (86 in 334), 24.3% (81 in 334), and 24.9% (83 in 334) patients, respectively. Patients in the Quartile 4 group had higher triglycerides, total cholesterol, and LDL-cholesterol levels and lower HDL-cholesterol levels. Patients in the Quartile 4 group had higher baseline fasting blood glucose compared to other groups, but the differences between the groups were not statistically significant. The histories of smoking and hypertension were similar among the four groups. Baseline age, BMI, duration of diabetes, HbA1c, eGFR, urinary protein excretion, hemoglobin, plasma albumin, insulin use, RAAS inhibitor use, statin use, and dyslipidemia were comparable in different groups (Table 1).

Regarding pathologic characteristics, patients in the Quartile 4 group had more severe arterial hyaline compared to other groups, but the differences between the groups were not statistically significant. The degree of glomerular lesions, interstitial fibrosis and tubular atrophy, interstitial inflammation, and arteriosclerosis were comparable in the different groups (Table 2).

### RC and renal outcome of DN

**Fig. 2:**
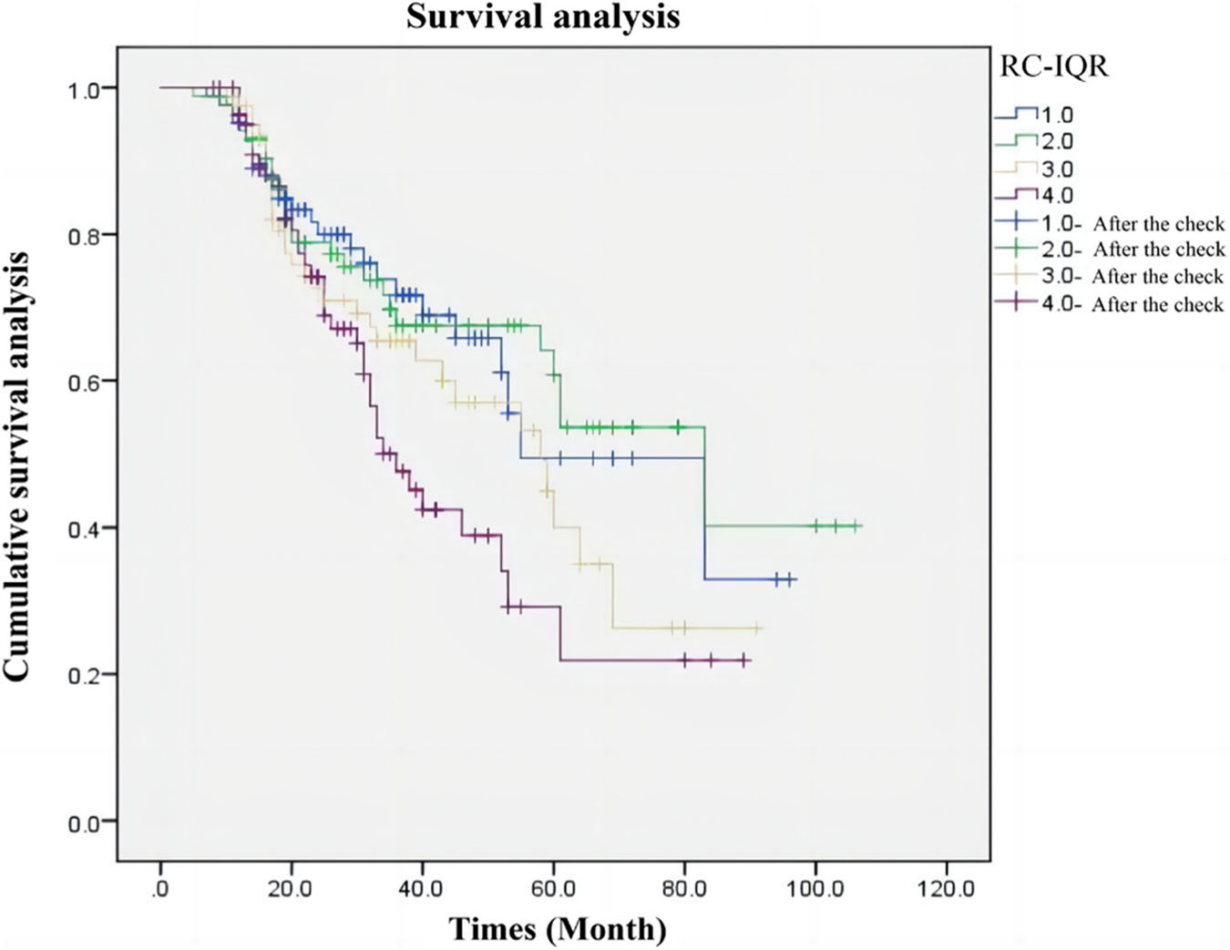
Renal survival rate between patients in four groups by Kaplan–Meier. The event-free survival for end stage kidney diseases (log-rank test, p = 0.033)

**Fig. 3:**
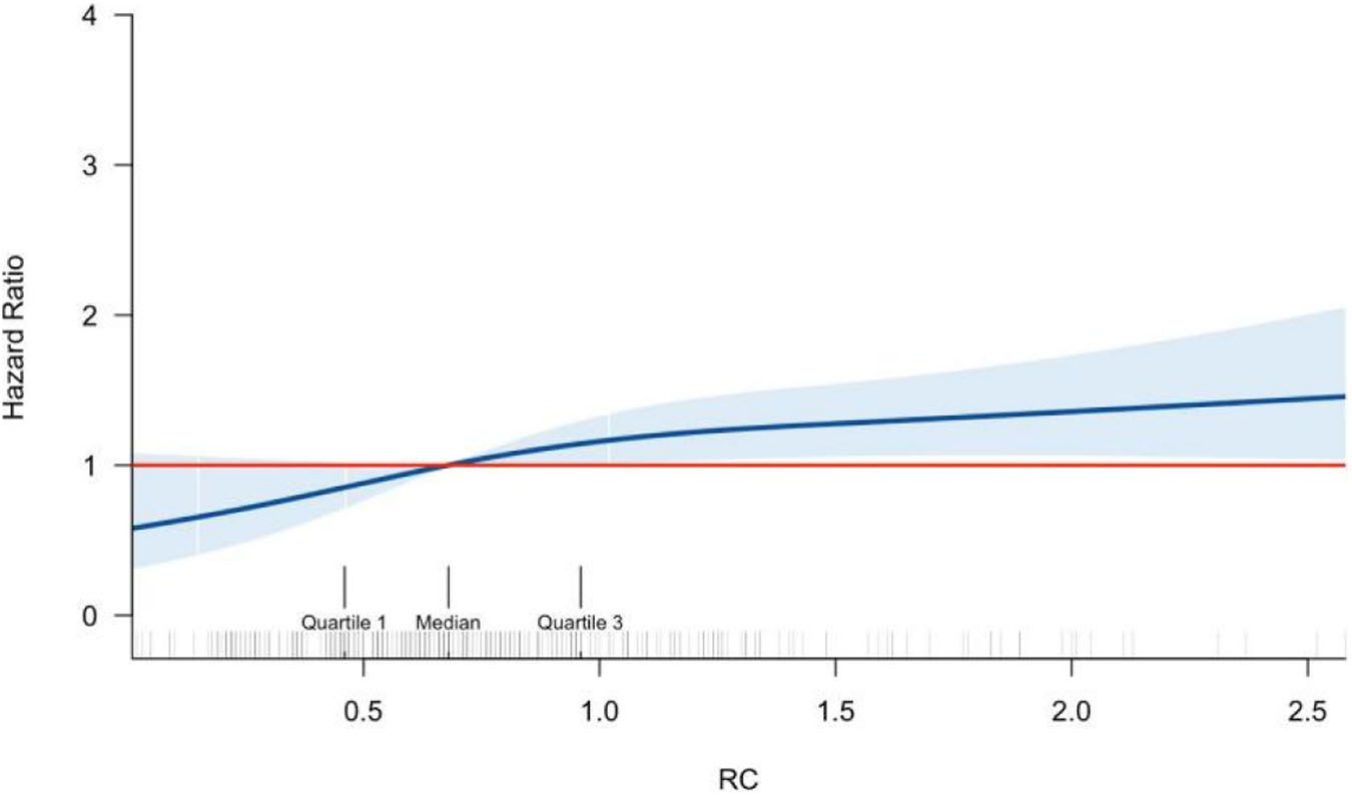
Dose-response relationships of baseline remnant cholesterol with incidence of ESKD. Restricted cubic spline regression model was conducted using 3 knots. The blue line and blue area represent the hazard radios and the 95% confidence intervals for the spline model

At the end of the study, during a median follow-up of 27 (17–43) months, a total of 121 (36.2%) patients reached kidney outcomes. Patients in the Quartile 4 group had a shorter median renal survival time [34.0 (26.4–41.6) vs. 55.0 (29.8–80.2) months] than patients in the Quartile 1 group. The Kaplan‒Meier test indicated that patients in the quartile 4 group had worse cumulative kidney survival than those in the other groups (log-rank test, p = 0.033) (Fig. 2). For further study, we used the Cox proportional model. In univariate analysis, high RC was associated with worse renal outcomes, regardless of a continuous variable (HR = 1.223; 95% CI 1.037–1.442, p = 0.017) or categorical variable (HR = 1.784; 95% CI 1.073–2.966, p = 0.026). To limit confounding factors, first adjusting for age, sex, BMI, hypertension, smoking, HbA1c, history of insulin use, history of RAAS inhibitor use, history of statin use and dyslipidemia (Model 1), high RC levels were still associated with a higher risk of ESKD in both continuous (HR = 1.489; 95% CI 1.138–1.949, p = 0.004) and categorical RC (HR = 3.323; 95% CI 1.489–7.418, p = 0.003). After further correcting for eGFR and urinary protein excretion (Model 2), higher RC levels were still significantly associated with a higher risk of progression to ESKD in patients with T2DM and biopsy-proven DN in continuous (HR = 1.440; 95% CI 1.081–1.917, p = 0.013) and categorical RC (HR = 3.101; 95% CI 1.377- 6.987, p = 0.006). To account for the effect of pathology type, we corrected for RPS and IFTA, which usually have a greater impact on nephropathy, and in addition, we included arteriolar hyalinosis considering that it may be associated with RC (model 3). The findings were consistent with the previous ones, with high RC levels being associated with higher risk of progression to ESKD in both continuous (HR = 1.439; 95% CI 1.080–1.917, p = 0.013) and categorical studies (HR = 3.351; 95% CI 1.452 – 7.735, p = 0.005) (Table 3). Figure 3 showed the dose-response relationship between baseline RC and the risk of progression to ESKD.

**Table 3:** Univariate and multivariate analysis for the association between remnant cholesterol and renal outcome

Parameter	Plasma RC, median (range) (pg/ml)	Hazard ratios (95% confidence interval) & *p* value			
		Unadjusted	Model 1	Model 2	Model 3
Per 1 SD RC	0.88 ± 0.80	1.223 (1.037–1.442) P = 0.017	1.489 (1.138–1.949) P = 0.004	1.440 (1.081–1.917) P = 0.013	1.439 (1.080–1.917) P = 0.013
Quartile 1	0.29 (0.21–0.42)	Reference	Reference	Reference	Reference
Quartile 2	0.59 (0.53–0.65)	0.923 (0.534–1.594) P = 0.773	1.023 (0.447–2.343) P = 0.956	1.192 (0.513- 2.771) P = 0.683	1.375 (0.562 –3.367) P = 0.486
Quartile 3	0.82 (0.76–0.92)	1.335 (0.790–2.254) P = 0.280	1.757 (0.802–3.847) P = 0.159	1.598 (0.704–3.628) P = 0.263	1.531 (0.627 –3.742) P = 0.350
Quartile 4	1.34 (1.17–1.89)	1.784 (1.073–2.966) P = 0.026	3.323 (1.489–7.418) P = 0.003	3.101 (1.377- 6.987) P = 0.006	3.351 (1.452 –7.735) P = 0.005

Unadjusted: univariate analysis

Model 1: adjusted for gender, age, body mass index, smoking, hypertension, HbA1c, dyslipidemia, RAAS inhibition use, insulin use, and statin use

Model 2: Model 1 further adjusted for eGFR and proteinuria

Model 3: Model 2 further adjusted for pathological features

## Discussion

This cohort study explored the longitudinal correlation between baseline remnant cholesterol and the progression to ESKD in patients with T2DM and biopsy-proven DN. Remnant cholesterol was a risk factor for DN progression to ESKD, independent of the traditional risk factors for ESKD, insulin use, RSSA inhibitor use, and statin use. In addition, patients with higher RC levels tended to have higher triglyceride, total cholesterol, and LDL-cholesterol levels, and lower HDL-cholesterol levels.

The previous study showed that higher remnant cholesterol is independently associated with an increased risk of prevalent CKD in a general middle-aged and elderly Chinese population [10]. Other studies showed that remnant cholesterol is independently associated with not only the risk of DN in T2DM patients [11] but also with the risk of the progression of DN and DR in T1DM patients [12]. Moreover, patients with higher remnant cholesterol levels tended to have higher triglyceride, total cholesterol, and LDL-cholesterol levels and lower HDL-cholesterol levels, regardless of general population, T2DM or T1DM patients. This is consistent with our study results, in which remnant cholesterol can be used as a biomarker to predict kidney outcomes in patients with T2DM and DN.

Previous studies have indicated that remnant cholesterol is an independent predictor of new-onset diabetes in the general population and kidney transplant recipients [18–20]. One of these studies found that the association between remnant cholesterol and new-onset diabetes may be mediated through insulin resistance and pro-inflammatory status [18]. Ohnishi et al. and Funada et al. reported the correlation between remnant cholesterol and insulin resistance [21, 22]. Moreover, the association between insulin resistance and the progression of DN has been reported in many clinical studies [23–25]. Numerous in vivo and in vitro experiments [26] have demonstrated that the pathophysiology of insulin resistance contributes to renal injury, including insulin resistance, which can contribute to the progression of glomerular hypertension and hyperfiltration by mediating an increase in vascular nitric oxide (NO) and transforming growth factor β1 (TGF-β1), higher salt sensitivity via upregulation of sodium-glucose cotransporters, endothelial dysfunction, and proteinuria via increased adipokine levels, activation of the TGF-β1/TGF-β receptor system and enhancement of profibrotic and pro-oxidant effects in glomerular cells via elevated leptin levels.

In addition, previous studies [18, 27] have observed a strong association between high remnant cholesterol and low-grade systemic inflammation, as evidenced by elevated C-reactive protein (CRP) and white blood cells (WBCs), suggesting that high remnant cholesterol is often accompanied by a proinflammatory state. In the Chronic Renal Insufficiency Cohort (CRIC) study [28], biomarkers of inflammation (IL-1β, IL-1 receptor antagonists, IL-6, TNF-α, CRP, and fibrinogen) were negatively associated with renal function and positively associated with proteinuria. Recently, low-grade chronic inflammation has also been shown to contribute to the progression of diabetic kidney disease, and several inflammatory biomarkers have been reported as prognostic markers to risk-stratify patients for disease progression and all-cause mortality [29]. However, whether remnant cholesterol contributes to DN progression through the mechanism of insulin resistance and low-grade system inflammation remains to be further investigated.

Furthermore, we found that remnant cholesterol and arteriolar hyalinosis may be related. Arteriolar hyalinosis, a characteristic pathological change in diabetes, has also been suggested as a risk factor for rapid decline in GFR. Due to the sample size limitation and the specificity of the study population, the results need to be validated in a larger diabetic population to explore whether residual cholesterol could have a role in the progression of diabetic nephropathy by affecting renal arteriolar hyalinization.

Moreover, another possible reason for DN progression is the atherogenic effect of remnant cholesterol. Numerous studies have shown that higher remnant cholesterol is associated with a higher risk of cardiovascular events and cardiovascular death in the general population and T2DM patients [7–9, 30]. Remnant cholesterol, the cholesterol component of triglyceride-rich lipoproteins (TRLs), is extremely atherogenic. Triglycerides, but not cholesterol, can be degraded in tissues or cells. TRLs cross the arterial wall where triglycerides are degraded and remnant cholesterol is deposited in the arterial wall, which in turn is taken up by macrophages or smooth muscle, generating foam cells and ultimately leading to the formation of atherosclerotic plaques [30]. Although, there is a common mechanism between microvascular and macrovascular complications of diabetes. Whether this is the reason for the further deterioration of renal function in DN due to remnant cholesterol needs to be further verified.

The results of this study also provide new ideas and perspectives for the lipid management of DN. First, clinicians can assess the risk of progression to ESKD in DN patients based on remnant cholesterol. According to our results, clinicians should pay more attention to patients with remnant cholesterol >1.02 mmol/L and take more aggressive lipid-lowering therapy to slow the progression of DN. Second, according to the recommendations of the 2013 KDIGO Clinical Practice Guidelines for Lipid Management in CKD [31], clinicians should regularly monitor patients’ triglycerides, total cholesterol, LDL-cholesterol, and HDL-cholesterol and assess patients’ risk of cardiovascular events based on LDL-c levels. Recently, several studies have found that remnant cholesterol may be a better marker than LDL-c for predicting cardiovascular outcomes in patients with T2DM [30]. Then, attempts should be made to incorporate remnant cholesterol to participate in the lipid management of DN patients to help decrease the incidence of cardiovascular events and death. However, it is worth noting that the optimal value of remnant cholesterol to guide lipid management in DN patients still needs to be determined in more and larger studies.

Up to now, statins are used to lower plasma LDL-c concentrations [32] and peroxisome proliferator–activated receptor (PPAR) agonizts are used to lower plasma triglyceride (TG) concentrations [33–35]. However, the use of first generation PPARα agonist (fibrate) has been limited by its side effects, including elevations in serum creatinine and homocysteine. Recently, in a randomized, placebo-controlled, double-blind, parallel-group phase 2 trial [36] enrolling statin-treated patients with hypertriglyceridemia, it was observed that a novel second-generation PPARα agonist (pemafibrate) is effective, safe, and well-tolerated for the reduction of TG, and also for the reduction of apoB48, apoCIII, and remnant cholesterol concentrations. This provides a new possibility and option to lower plasma triglycerides and remnant cholesterol concentrations. Unfortunately, there are no studies on the effects of pemafibrate on cardiovascular events and kidney outcomes. Therefore, further validation of the heart and kidney benefits necessitates additional real-world studies.

There are also some limitations in this study. First, we did not follow up with the patients’ lipids over time and dynamically and therefore could not observe the relationship between remnant cholesterol variability and the progression of DN to ESKD. Second, the outcomes of this study lacked data on cardiovascular events and deaths, higher RC levels have been shown to be associated with a high risk of cardiovascular and non-cardiovascular deaths, with an unknown association with the risk of cancer death. Third, this is a retrospective cohort study. Thus, selective bias is inevitable. However, given the overall matched baseline clinicopathological variables between the different groups and the use of Cox analysis, the results were generally reliable. Fourth, the sample size was limited, with only 334 patients included. What’s more, as a drug that affects both residual cholesterol and renal prognosis, the use of SGLT2i was not taken into account and may have influenced the analysis results. But considering that the drug was not highly utilized in China at that time, it had little impact on the reliability of the results. Finally, the results may be only applicable to patients with T2DM and biopsy-proven DN, not to patients with DKD or diabetes, and CKD.

## Conclusion

In conclusion, this longitudinal cohort study highlights the role of remnant cholesterol in patients with T2DM and biopsy-proven DN, extending previous knowledge. We showed that by calculating remnant cholesterol from standard lipid profiles, the progression of DN can be predicted independently of several clinically meaningful risk factors. The increasing burden of diabetes and its complications is concerning, and therefore, an urgent need for more efficient treatment strategies exists. Whether lowering remnant cholesterol could translate into a reduction in microvascular complications of diabetes remains to be clarified, and for that purpose, it needs to be assessed whether the associations we present in this observational study are causal or not.
